# Long-term low-dose ketoconazole treatment in bilateral macronodular adrenal hyperplasia

**DOI:** 10.1530/EDM-14-0083

**Published:** 2014-12-01

**Authors:** Sophie Comte-Perret, Anne Zanchi, Fulgencio Gomez

**Affiliations:** 1 Service of Endocrinology Diabetology and Metabolism, Department of Medicine, CHUV-University Hospital, Lausanne, CH-1011, Switzerland

## Abstract

Medical therapy for Cushing's syndrome due to bilateral macronodular adrenal hyperplasia (BMAH) is generally administered for a limited time before surgery. Aberrant receptors antagonists show inconsistent efficacy in the long run to prevent adrenalectomy. We present a patient with BMAH, treated for 10 years with low doses of ketoconazole to control cortisol secretion. A 48-year-old woman presented with headaches and hypertension. Investigations showed the following: no clinical signs of Cushing's syndrome; enlarged lobulated adrenals; normal creatinine, potassium, and aldosterone; normal urinary aldosterone and metanephrines; elevated urinary free cortisol and steroid metabolites; and suppressed plasma renin activity and ACTH. A screening protocol for aberrant adrenal receptors failed to show any illegitimate hormone dependence. Ketoconazole caused rapid normalisation of cortisol and ACTH that persists over 10 years on treatment, while adrenals show no change in shape or size. Ketoconazole decreases cortisol in patients with Cushing's syndrome, and may prevent adrenal overgrowth. Steroid secretion in BMAH is inefficient as compared with normal adrenals or secreting tumours and can be controlled with low, well-tolerated doses of ketoconazole, as an alternative to surgery.

**Learning points:**

- Enlarged, macronodular adrenals are often incidentally found during the investigation of hypertension in patients harboring BMAH. Although laboratory findings include low ACTH and elevated cortisol, the majority of patients do not display cushingoid features.
- Bilateral adrenalectomy, followed by life-long steroid replacement, is the usual treatment of this benign condition, and alternative medical therapy is sought. Therapy based on aberrant adrenal receptors gives disappointing results, and inhibitors of steroidogenesis are not always well tolerated.
- However, ketoconazole at low, well-tolerated doses appeared appropriate to control adrenal steroid secretion indefinitely, while preventing adrenal overgrowth. This treatment probably constitutes the most convenient long-term alternative to surgery.

## Background

Hypercorticism with low plasma adrenocorticotrophin (ACTH) and cell proliferation in patients with macronodular adrenal hyperplasia is ascribed to intrinsic adrenal changes, that include the frequent expression of aberrant G-protein-coupled receptors in the membranes of steroidogenic cells that are stimulated by a variety of circulating ligands (1) (2), and a possible paracrine effect of corticotropin produced by the same hyperplastic adrenal tissue (3). In addition, germline and somatic inactivating mutations of a putative tumour-suppressor gene, armadillo repeat containing 5 (*ARMC5*), were recently found in the adrenal glands of about half of patients with bilateral macronodular adrenal hyperplasia (BMAH) (4), and an autosomal dominant mode of inheritance was further characterised (5). Therefore, the terms ‘primary’ or merely ‘bilateral’ macronodular adrenal hyperplasia (and thus the acronyms PMAH or BMAH) could be most appropriate to qualify the syndrome of macronodular adrenal hyperplasia with hypercorticism, which was formerly termed ‘ACTH-independent’ (3) (6).

At the present time, there are no guidelines for the management of this rare condition and, notwithstanding its benign nature, most patients receiving medical therapy for BMAH finally resolve to undergo surgical adrenalectomy (7). Medical therapy based on aberrant adrenal receptors give inconsistent results for the long-term control of steroid secretion, either with receptor antagonists (8) or with somatostatin analogues that suppress illicit ligands (9) (10). On the other hand, prolonged treatment with inhibitors of steroidogenesis can be hampered by drug intolerance, yet a lifelong treatment would be required to avoid surgery. In fact, reports on prolonged inhibition of steroidogenesis in BMAH are scanty. Trilostane, a 3β-hydroxysteroid dehydrogenase inhibitor, did not prevent continuing adrenal growth during a 4-year treatment period, despite partial suppression of cortisol production per adrenal mass with clinical improvement (11). Metyrapone, an 11β-hydroxylase inhibitor, has long been used to treat severe forms of Cushing's syndrome, but may cause hypoadrenalism and hyperandrogenism as the most common side-effects (12). However, it was successfully used in two Japanese patients with BMAH, including one woman treated for 7 years and presenting no hirsutism (13) (14). Ketoconazole is an antifungal agent that also reduces adrenal and gonadal steroid production via the inhibition of several steroidogenic enzymes (11β-hydroxylase, 17α-hydroxylase and 18-hydroxylase), as well as cholesterol side-chain cleavage (15), and has been widely used as a palliative treatment or as an alternative to surgery in different forms of Cushing's syndrome, at doses as high as 1200 mg/day. As this drug does not induce adrenal hyperandrogenism, it could be an interesting option in women. Many cases of BMAH are incidentally discovered, with little or no cushingoid features despite markedly enlarged adrenals (1) (2), suggesting relatively inefficient cortisol secretion by the macronodular tissue, which might respond to lower doses of ketoconazole for steroid control.

To our knowledge, only one case of successful treatment with ketoconazole has been reported in BMAH, during a 1-year therapy until adrenalectomy was performed, at doses that were not mentioned (10). We present a patient with BMAH without any evidence of illegitimate hormone dependence, treated successfully during 10 years with well-tolerated low doses of ketoconazole.

## Case presentation

**Figure 1:**
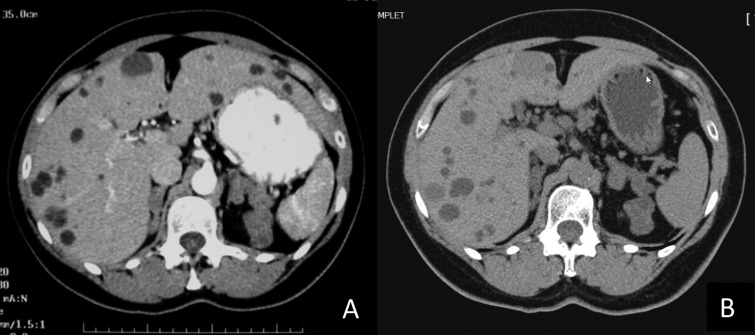
Abdominal CT-scan at baseline (A) and after 10 years on low-dose ketoconazole (B). Multi-lobulated bilateral adrenal masses are observed, which remain unchanged in morphology after 10 years of treatment. Volume was calculated using the ellipsoid formula π abc/6 (ml): (a) anterior–posterior, (b) transversal, and (c) cranial–caudal maximal diameters. (A) Right adrenal 12.9 ml and left adrenal 20.5 ml. (B) Right adrenal 14.7 ml and left adrenal 20.9 ml. Hepatic cysts are due to familial polycystic liver disease.

A 48-year-old woman presented with sudden headaches and new-onset marked hypertension. She had no signs of Cushing's syndrome. She was known for familial (mother and brother) asymptomatic polycystic liver disease without kidney involvement. Abdominal ultrasound study showed normal kidneys, numerous hepatic cysts and unexpected large multi-lobulated bilateral adrenal masses, confirmed on CT-scan (Fig. 1A). Laboratory studies showed normal serum creatinine and urinary metanephrines; low-normal serum potassium (3.85 mmol/l; normal range 3.50–5.20 mmol/l), normal plasma aldosterone concentration (62 ng/l; 29–76 ng/l), normal urine aldosterone (4.1 μg/24 h; 1.0–10.0 μg/24 h) and suppressed plasma renin activity (<0.05 μg/l per h; 0.20–2.00); elevated urine tetrahydro-metabolites of 11-deoxycortisol (THS, 396 μg/24 h; 10–109 μg/24 h), corticosterone (THB, 509 μg/24 h; 26–262 μg/24 h), cortisone (THE, 6877 μg/24 h; 727–3815 μg/24 h) and cortisol (THF, 3031 μg/24 h; 458–1907 μg/24 h); elevated urine free cortisol (349 nmol/24 h; 55–248 nmol/24 h); normal plasma AM cortisol (309 nmol/l; 200–700 nmol/l at 0800 h) and slightly elevated PM cortisol (365 nmol/l; 60–300 nmol/l, at 1700 h), low ACTH (5 ng/l; 10–60 ng/l) and suppressed DHEAS (<0.5 μmol/l; 1.6–7.0 μmol/l).

## Investigation

Repeat measurements showed undetectable ACTH and DHEAS and elevated plasma cortisol. A cosyntropin test (0.25 mg as i.v. bolus) elicited an intense plasma cortisol response (from basal 444 nmol/l to 1147 nmol/l, +158%, at 60 min) and urine free cortisol response (2776 nmol/24 h on the test day and 2036 nmol/24 h on the day after), indicating high adrenal sensitivity to ACTH.

BMAH was diagnosed and a screening protocol for aberrant adrenal receptors was performed according to Lacroix *et al*. (16). No significant increase in plasma cortisol (less than +25% of basal value) was observed after a mixed meal of 500 kcal, 40% carbohydrates, 35% lipids, and 25% proteins (−18% of basal value), and orthostatism (+11%), metoclopramide 20 mg p.o. (+8%), glucagon 1 mg i.v. bolus (+5%), gonadotrophin-releasing hormone 0.1 mg i.v. bolus (+19%), follicle-stimulating hormone 300 IU i.m. (+10%), human chorionic gonadotrophin 1000 IU i.m. (+4%), or thyrotrophin-releasing hormone 0.2 mg i.v. bolus (+2%), and no decrease was observed after propranolol 80 mg p.o. (+28%). ACTH was undetectable during these tests. It was concluded that there were no aberrant receptors sensitive to the hormonal systems explored.

## Treatment

After appropriate information and discussion with the patient, medical therapy was preferred to bilateral adrenalectomy and life-long steroid replacement. Medical inhibition of adrenal steroidogenesis with oral ketoconazole was initiated, at 200 mg/day and then 400 mg/day, with a rapid decrease in urine free cortisol and increase in plasma ACTH to normal. Blood pressure was rapidly normalised and eventually required only small doses of spironolactone (25 mg/day) and metoprolol (100 mg/day).

## Outcome and follow-up

**Figure 2:**
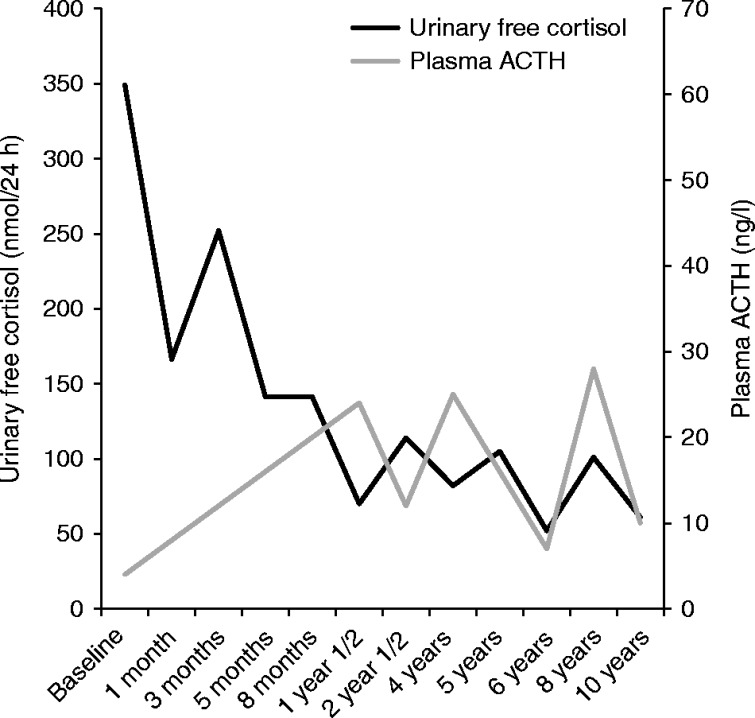
Urinary free cortisol and plasma ACTH after starting ketoconazole. Normal values of urinary free cortisol are shown with the shaded area.

Cortisol and ACTH have remained normal throughout more than 10 years on treatment (Fig. 2); however, DHEAS remained undetectable. Bone mineral density (BMD) at diagnosis showed hip-cortical osteopenia and normal lumbar spine trabecular density *T*-scores. Control BMD at 7 years of treatment showed an unchanged cortical value, corresponding to a relative increase in BMD with a *Z*-score at percentile 30–40, and a normal trabecular density with an unchanged *Z*-score at percentile 50. Ketoconazole is well tolerated, with no signs of drug toxicity on repeat liver enzymes, creatinine levels and electrocardiogram. Repeat CT-scan studies show no significant changes in macronodular adrenals and liver cysts (Fig. 1B). Adrenal glands were measured on CT scans and volume was calculated using the ellipsoid formula π abc/6 (ml): (a) anterior–posterior, (b) transversal, and (c) cranial–caudal maximal diameters. Control at 10 years showed a minimal increase of 1.8 ml in the right adrenal and of 0.4 ml in the left gland.

## Discussion

Although the natural growth of adrenal masses in BMAH has not been widely documented, studies in familial BMAH have identified adrenal nodules in asymptomatic young relatives with germline mutations in ARMC5 (4). Careful measurements of adrenal volume on serial CT scans performed in one patient showed a progressive increase in the adrenal volume, which was not slowed with chemical single-enzyme inhibition of steroidogenesis despite adequate cortisol suppression (11). These observations confirm the generally admitted dissociation between adrenal growth and cortisol secretion and suggest slow mass progression before excess cortisol becomes apparent. Enlarged macronodular adrenals are often unexpectedly found during the investigation of hypertension (1) (2), as in the patient described here. Hypertension in our patient was largely due to excess adrenocortical secretion, as corroborated by the response to medical inhibition of adrenal steroidogenesis. Precursor mineralocorticoids probably contributed to hypertension, as suggested by elevated urinary metabolites and low aldosterone and renin levels observed in our patient. Predominant co-secretion of precursor mineralocorticoids, and low aldosterone and renin levels, were previously reported in Japanese patients with BMAH, who had slightly elevated cortisol levels (17) (18) (19). The high sensitivity to corticotropin displayed by our patient despite prolonged suppression of pituitary ACTH is consistent with a paracrine priming effect on steroidogenic cells, exerted by ACTH produced *in situ* within the same macronodular adrenals (3). Suppressed DHEAS at presentation may be ascribed to low circulating ACTH, but persistent suppressed DHEAS despite normal ACTH during treatment could be a direct effect of ketoconazole (suppression of 17α-hydroxylase) or the result of intrinsic changes within the adrenal nodules, similar to those of testicular adrenal rest tissue in patients with congenital adrenal hyperplasia (increased 3β-hydroxysteroid dehydrogenase activity) (20).

Medical therapy for Cushing's syndrome due to BMAH is generally administered for a limited time, before adrenalectomy and life-long steroid replacement. Clinicians may prefer to treat by bilateral adrenalectomy, considering that patients on medical therapy could stay mildly hypercortisolic or have fluctuating levels of cortisol. However, our patient presented constantly normal urine free cortisol and plasma ACTH during treatment, never developed a cushingoid appearance and showed a stable or an improved BMD. This evolution speaks against a significant chronic hypercortisolism. Furthermore, safety controls have remained normal during this long observation. Thus, although not a common practice, indefinite medical therapy with the purpose to avoid surgery can be a convenient and safe approach in some patients with BMAH.

Ketoconazole decreases cortisol secretion in patients with Cushing's syndrome in general, on a dose range of 400–1200 mg/day (21). In a large retrospective study in patients with Cushing's disease, a median final dose of 600 mg/day ketoconazole was required for the control of cortisol secretion (22). On the contrary, the BMAH patient here described, required doses that were in the lowest range described to control Cushing's disease. Although in that retrospective study, the dose did not predict the response, the small doses required by our patient despite a particularly large adrenal size indicate a relatively inefficient cortisol secretion by the BMAH nodules, as compared with adrenal glands that are intrinsically normal but are submitted to intense ACTH overstimulation.

Our observations also suggest that these low doses may have prevented adrenal overgrowth. Indeed, the increase in adrenal size observed at 10 years was minimal as compared with the massive enlargement of 22.6 and 35.4 ml described under trilostane at 7 years (11), and probably was within the error of the method of measurement given the complex shape of the glands.

Ketoconazole is metabolised into inactive compounds, primarily by the liver, and metabolites are excreted mostly in the faeces, with very little excretion into the urine. Renal impairment does not seem to cause accumulation of the drug, but hepatic insufficiency contraindicates its use (15). Associating other drugs should be done with caution, because ketoconazole inhibits microsomal CYP3A4 in the liver and gastrointestinal tract, and may hamper drug metabolism. Most common side effects at the doses used for fungal infection (200–400 mg/day) are gastrointestinal, pruritus and liver dysfunction (15). A meta-analysis reported an incidence of ketoconazole-induced hepatotoxicity of 3.6–4.2%; however, in this study hepatotoxicity was defined as an increase in alanine aminotransferase (ALT) and/or in total bilirubin (TB) >1× upper normal limit (u.n.l.) on two consecutive measurements, or an increase in ALT >2× u.n.l. and/or TB >1× u.n.l. on one measurement (23), and the actual incidence of severe hepatotoxicity was not reported. The retrospective study on ketoconazole in Cushing's disease reported a mild (<5× u.n.l) and a major (>5× u.n.l) increase in liver enzymes in 13.5 and 2.5% respectively (22). This drug should nevertheless be used carefully and liver enzymes measured regularly. In July 2013, the US Food and Drug Administration and the European Medicines Agency (EMA) proposed to limit the use of oral ketoconazole in fungal infections, because the estimated risks overweighed the benefits, mainly due to liver toxicity but also due to adrenal insufficiency and interaction with other drugs (24) (25). However, these agencies did not give recommendations about its use in Cushing's syndrome, and the EMA states that they are aware that ketoconazole is being used off-label in such cases. Oral ketoconazole has been used at high doses to control cortisol secretion in Cushing's disease, in some instances for more than 2 years (21), and in non-treatable ectopic ACTH syndrome.

Our observations suggest that ketoconazole, at well-tolerated low dose, can control steroid secretion in BMAH indefinitely, while preventing adrenal overgrowth. Therefore, and considering the benign nature of BMAH, this medical treatment is a convenient alternative to surgery, for patients who show good response and who can be appropriately followed up. Regular controls are required not only for efficacy and drug toxicity but also to detect subclinical chronic cortisol excess.

## Patient consent

We confirm that written informed consent was obtained from patient for publication of this case report and associated images.

## Author contribution statement

S Comte-Perret and F Gomez were directly involved in the management of this patient. This manuscript has been contributed, seen and approved by all the authors, who have participated sufficiently in the work to take public responsibility for its content.
